# Sex Worker Community-led Interventions Interrupt Sexually Transmitted Infection/Human Immunodeficiency Virus Transmission and Improve Human Immunodeficiency Virus Cascade Outcomes: A Program Review from South India

**DOI:** 10.1097/OLQ.0000000000001020

**Published:** 2019-07-11

**Authors:** Sushena Reza-Paul, Richard Steen, Raviprakash Maiya, Robert Lorway, Teodora Elvira Wi, Tisha Wheeler, Gina Dallabetta

**Affiliations:** From the * Ashodaya Samithi, Mysore, Karnataka, India;; † Department of Community Health Sciences, University of Manitoba, Winnipeg, Canada;; ‡ Department of Public Health, Erasmus MC, University Medical Center Rotterdam, Rotterdam, The Netherlands;; § Department of Reproductive Health and Research, World Health Organization, Geneva, Switzerland;; ¶ USAID, Arlington, VA; and; ∥ The Bill & Melinda Gates Foundation, Washington, DC

## Abstract

Ashodaya Samithi, an organization run by and for female, male, and transgender sex workers in Mysore, India, has worked since 2004 to prevent sexually transmitted infection (STI)/human immunodeficiency virus (HIV) transmission and improve HIV cascade outcomes. We reviewed published and programmatic data, including measures of coverage, uptake, utilization and retention, and relate STI/HIV outcomes to evolving phases of community mobilization. Early interventions designed “for” sex workers mapped areas of sex work and reached half the sex workers in Mysore with condoms and STI services. By late 2005, when Ashodaya Samithi registered as a community-based organization, interventions were implemented “with” sex workers as active partners. Microplanning was introduced to enable peer educators to better organize and monitor their outreach work to reach full coverage. By 2008, programs were run “by” sex workers, with active community decision making. Program data show complete coverage of community outreach and greater than 90% clinic attendance for quarterly checkups by 2010. Reported condom use with last occasional client increased from 65% to 90%. Surveys documented halving of HIV and syphilis prevalence between 2004 and 2009, while gonorrhoea declined by 80%. Between 2005 and 2013, clinic checkups tripled, whereas the number of STIs requiring treatment declined by 99%. New HIV infections also declined, and Ashodaya achieved strong cascade outcomes for HIV testing, antiretroviral treatment linkage, and retention. Program performance dropped markedly during several periods of interrupted funding, then rebounded when restored. Ashodaya appear to have achieved rapid STI/HIV control with community-led approaches including microplanning. Available data support near elimination of curable STIs and optimal cascade outcomes.

## Abstract 2

A review of a community-led sex worker program in 2 districts of South India that has achieved near elimination of curable sexually transmitted infections and optimal human immunodeficiency virus cascade outcomes.

Supplemental digital content is available in the text.

The centrality of sex work in the epidemiology of HIV and other STIs, and of sex workers as critical partners in prevention and epidemic control efforts, is increasingly recognized.^1,2^ In recent years, however, much available funding for sex worker programming has prioritized a limited range of interventions, often closely tied to HIV treatment cascade targets.^2^ We review the experience of a sex worker community in Southern India that has succeeded in both interrupting STI/HIV transmission and optimizing HIV cascade outcomes.

Water analogies are commonly used in public health to describe transmission streams and to visualize intervention steps. Cascade analysis focused on HIV testing and antiretroviral treatment (ART) linkage, with widely adopted 95–95–95 targets, derive from a general population approach to “treatment as prevention.”^1^ If 95% of a population knows their HIV status and 95% of those living with HIV are started and retained on ART, with 95% suppression of viral load, onward HIV transmission should be averted for 87% of that population.

Upstream-to-downstream models argue that STI epidemics can be controlled by intervening effectively with small subgroups of “key populations”—sex workers, men who have sex with men, transgender persons, injecting drug users—who are disproportionately affected. High rates of partner change in sex work potently drive transmission within and beyond “upstream” networks, sustaining high prevalence “downstream” among the general population. Empirical data and modeling argue that effective targeting of such high-incidence sexual networks is necessary to achieve epidemic control.^3–6^

The combination of these 2 approaches is potentially powerful.^1,2,7^ But real-life implementation does not always proceed in synergistic or complementary ways. A country can achieve 95–95–95 and still miss most key populations—who make up only a few percent of the population and are often marginalized.^8^ Moreover, distortions can emerge when key population funding is based on program “yield” of HIV-positives. Human immunodeficiency virus testing may be pushed too early, too narrowly, too aggressively, alienating key populations, and driving them away from services. A Catch-22 may even result where only weak key population programs—by failing to control transmission—are able to deliver high numbers of new positives over time. There is scant attention to these issues, and little programmatic experience, described in the literature.^9^

Ashodaya Samithi, a sex worker-run community-based organization (CBO), provides a good example of how these 2 streams of HIV intervention efforts can flow together synergistically. Ashodaya's STI/HIV prevention interventions have been operational since 2004 to reduce STI/HIV transmission, and since 2008 to also maximize treatment access and outcomes. In addition to its core work with local female, male, and transgender sex worker communities, Ashodaya supports scale-up of community-based interventions elsewhere in India, and currently serves as a regional and global learning site.

We reviewed published and programmatic data from 2004 through 2018 to describe interventions and STI/HIV trends among sex workers in the context of Ashodaya program implementation. We analyzed outreach coverage data, trends from routine clinical screening, and survey data for evidence of STI/HIV declines. Ashodaya participated in the Avahan India AIDS Initiative, and contributed to development of the Avahan “common minimum program” (described elsewhere), which set standards for community-led structural interventions, outreach, clinical services, commodities, advocacy, and data and program management.^10^

## An Evolving Community-based Response to HIV and Other STIs

Ashodaya Samithi operates in Mysore and Mandya districts in the state of Karnataka, India. The Ashodaya community-led experience is examined over 3 periods—(1) start-up, (2) community consolidation, and (3) recent program interruption and recovery.

### Start-up Phase (2004–2008)

Interventions with Mysore sex workers began in 2004 when a public health team launched an HIV intervention project to increase condom use among sex workers. Most sex workers in Mysore at the time were street-based, soliciting clients at the central bus station and other “hotspots,” and using nearby hotels or lodges where rooms were rented for short periods. These early interventions “for” sex workers (led mainly by program staff and researchers) not only succeeded in increasing condom use and clinic visits but also built a level of trust within the sex worker community that facilitated their progressive engagement and involvement.^11^ Early support from Avahan enabled development of a targeted intervention model that was disseminated and adapted widely across India.^10^

Within a year, the program, increasingly implemented “with” sex workers, had mapped sex work areas and was reaching local sex workers with basic peer outreach, education on HIV and STIs, condom distribution, and STI screening and treatment interventions. The community became increasingly involved in decision making, whereas program staff adapted to new roles as facilitators, encouraging community engagement and participation.

The sex worker community formed its own organization, Ashodaya Samithi, in December 2005. By the third year of the program, more than 90% of female sex workers surveyed reported having been visited by a peer educator, attending the drop-in center and dedicated sexual health clinic, and accepting presumptive STI treatment.^11^ Interventions and services were also extended to neighboring Mandya district. In a step-by-step, participatory manner, the outreach team developed an early version of an outreach planning tool, which later matured into a microplanning approach, in use since 2008. This outreach planning, reflecting program aims to maximize scale, coverage, and quality, transferred outreach management to community members themselves, using routinely collected data, including annual population size estimates, for local decision making.

Some early successes of these efforts were partially captured in 2 cross-sectional surveys conducted 30 months apart in 2004 and 2006.^11^ Reported condom use at last sex by Mysore sex workers rose significantly from 65% to 90% with occasional clients, from 7% to 30% with regular partners, and from 53% to 66% with repeat clients. Syphilis prevalence declined from 25% to 12%, chlamydia from 11% to 5%, and gonorrhoea from 5% to 2%, trichomoniasis from 33% to 14%, all statistically significant. HIV prevalence initially remained stable (26% versus 24%) but detuned assay testing showed a significant decline in recent HIV infections. The HIV prevalence subsequently declined significantly (to 11% in 2009).^12^ These results may underestimate the true magnitude of change since interventions to meet the immediate needs of the community had begun 6 months before the baseline survey was conducted.

From the beginning, steps were taken to address the considerable violence faced by sex workers using community-led approaches. One of the first steps was to create a “safe space” or drop-in center where sex workers could meet, wash, relax, and interact with each other without fear of harassment. Project staff also became involved in crisis management, such as negotiating the release of sex workers after arrests and addressing violence from police, *goondas* (local thugs), clients, and boyfriends.^13^ In 2007, Ashodaya introduced a self-regulatory board whereby sex workers took the lead on combatting trafficking, collaborating with local authorities to prevent coercion and underage sex work. These community-led approaches increased community ownership and utilization of health services.^13–15^

Toward the end of this period (2008), Ashodaya began strengthening linkages to government ART services for sex workers living with HIV. A number of program innovations were developed to make HIV testing more acceptable and accessible, to link those testing positive to treatment and to support them to sustain high retention.^16^ Ashodaya formed an “accompanied referral” system whereby volunteers, often HIV-positive sex workers, accompanied consenting sex workers to HIV testing and ART clinics. These volunteers facilitated follow-up of those testing positive, whether sex workers or not. This help was appreciated by clinic staff and helped reduce stigma or discrimination toward sex workers visiting public hospitals. Through a public-private partnership model, Ashodaya started offering HIV testing at the drop-in center clinic. In 2009, an organization of HIV-positive sex workers (Ashraya) formed. Members actively addressed incidents of stigma and discrimination and conducted advocacy to improve services, addressing problems such as ART stock-outs and lack of CD4 count machine.

### Community Consolidation Phase (2009–2013)

By 2009, Ashodaya registered as a CBO with a democratically elected board of sex workers responsible for governance and decision making and was engaging with the wider community on a range of common issues. During this phase, programs were run “by” the community, which became empowered to effect change across a range of areas beyond STI/HIV prevention.^13^

During this period, Ashodaya made considerable progress in consolidating and improving the operational performance of its program. Microplanning was formally adopted, strengthened, and rolled out everywhere as Ashodaya's outreach strategy.^17^ The tool strengthened the capacity of peer educators in planning, implementing, and assessing their community work. Microplanning also boosted sex worker ownership of these community-based interventions.

**Figure 1:**
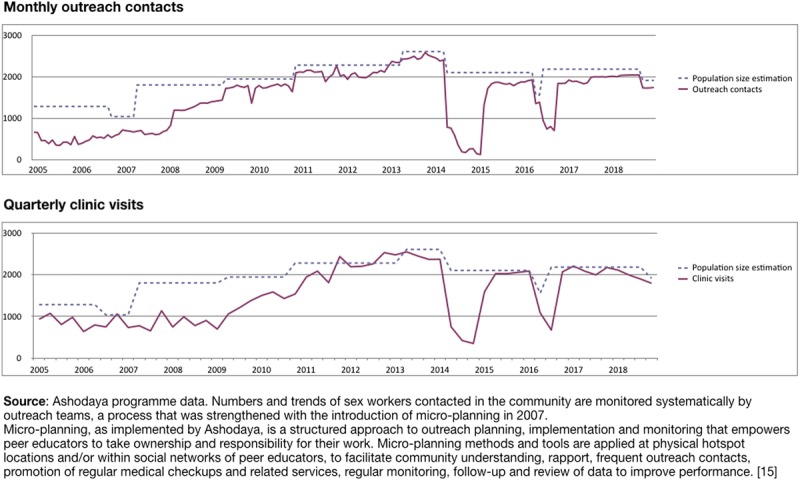
Unique monthly outreach contacts and quarterly clinic visits by population size estimates.

**Figure 2:**
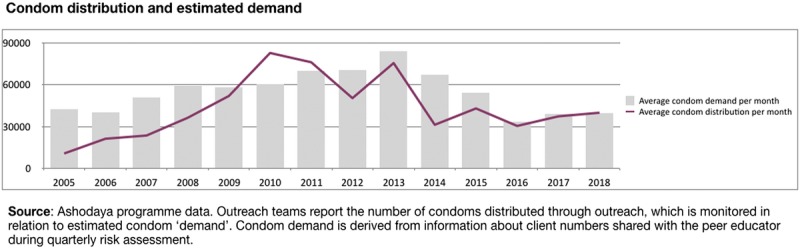
Condom distribution against estimated demand (based on client numbers).

**Figure 3:**
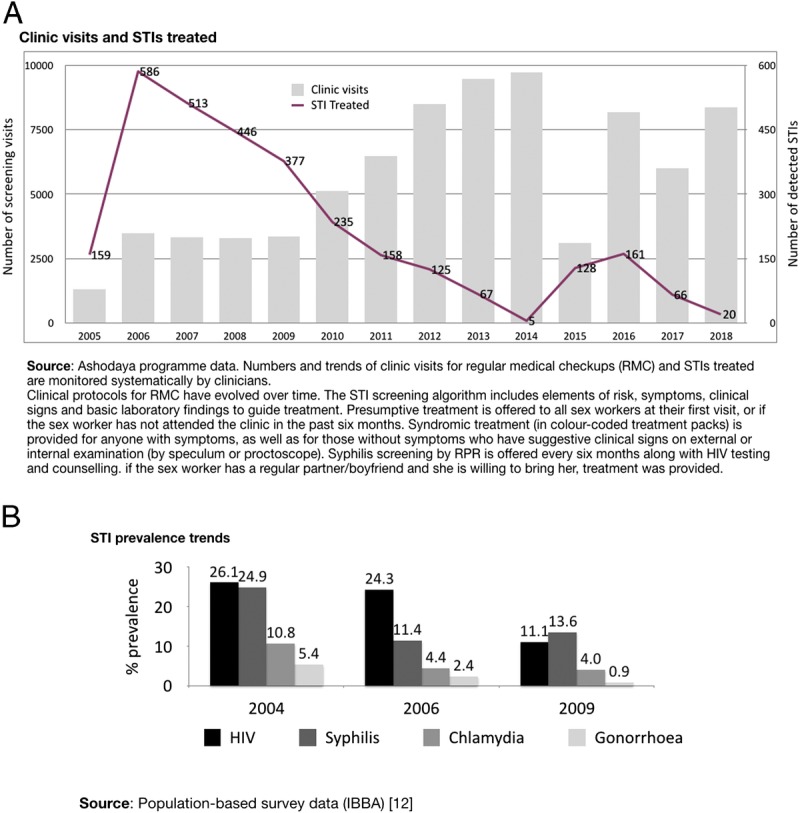
A, Data on clinic visits and symptomatic STIs treated. B, STI/HIV prevalence by survey.

By 2013, all key program indicators—monthly outreach contacts, condom distribution, quarterly clinic visits—were showing strong performance compared to population size estimates (Figs. 1 to 3). Coverage of community outreach reached “saturation” levels, with nearly all sex workers contacted at least monthly (unique identifiers were used to facilitate monitoring). During this period, sex workers also attended clinics quarterly for regular medical checkups (RMC), where they were offered STI screening and treatment, family planning, and other sexual and reproductive health services, following an occupational health model.^15,18^ Several clinical outcome measures were continuously monitored, and the number of STIs detected progressively declined, despite increasing clinic attendance (Fig. 3A).

Free condom distribution increased by 2009 to meet estimated need—based on reported client numbers and frequency of sex (Fig. 2). In response to the high burden of curable STIs, periodic presumptive treatment (PPT) was introduced in 2004, in consultation with community members. Comprising a single-dose treatment of azithromycin 1G and cefixime 400 mg, PPT was initially offered quarterly at regular checkups, regardless of STI symptoms, then tapered to 6-monthly after 2006 integrated bio-behavioral assessment results showed significant STI declines (Fig. 3B). After 2010, PPT was only offered to new sex workers at first visit or to those who had not attended clinic for 6 months. STI treatment based on symptoms and speculum examination findings is currently offered routinely at regular medical checkups (see STI algorithm in supplemental file, http://links.lww.com/OLQ/A386).

Program data validated by survey results argue that this combination of frequent outreach, condom distribution, and regular clinic attendance contributed to large reductions in preexisting STI burden and to slowing STI/HIV transmission. The STI prevalence declines measured over the first 2 years continued or stabilized (Fig. 3B), trends that were also seen in other districts of Karnataka where Avahan supported similar interventions.^12,19,20^ Community mobilization was found to be an independent factor in both gonorrhoea and chlamydia prevalence reductions.^21^

Routine clinic data enabled the program to monitor a steady decline in symptomatic STIs (Fig. 3A). Still, between 11% and 16% of sex workers seen for checkups from 2004 through 2008 had STI symptoms that required treatment (based on STI management algorithm, see supplemental file, http://links.lww.com/OLQ/A386).^18^ From 2009 to 2013, however, the period of more intensive outreach, condom distribution, and clinic checkups, this proportion fell from 5% to less than 1%. Between 2005 and 2013, the number of visits for regular medical checkups increased almost 3-fold, whereas the number of STIs requiring treatment declined by a factor of more than 100 (from 586 to 5). Despite these declining rates of symptomatic STIs, continued quarterly clinic attendance was promoted to stay healthy and to avail other sexual and reproductive health services.^22^ Rates of active syphilis (rapid plasma reagin ≥ 1:8, treponema pallidum hemagglutination assay confirmed) detected by routine screening every 6 months continued to decline, to 0.8% (11 of 1434) in 2012, and 0.04% (1 of 2116) in 2013 (Ashodaya program data).

### Recent Interruptions and Recovery (2014–2018)

In 2013, key populations programs in India, including those supported by Avahan, transitioned to government funding, with nationally standardized guidelines and targets.^23^ During the transition, Ashodaya retained most of its critical program elements, including community mobilization through peer outreach with microplanning, and community-led service delivery. Performance across priority program indicators continued to be strong. During the posttransition period, however, an extended interruption of funding by the government for targeted interventions, from May 2014 to February 2015, led to sharp drops in outreach (Fig. 1), condom distribution (Fig. 2) and routine checkups (Fig. 3A). This was followed by a surge in symptomatic STI cases at Ashodaya clinics (Fig. 3A). Ashodaya had previously documented temporary interruptions in outreach and service utilization in 2006, during a 3-month period of police harassment. In both cases, community efforts including strong advocacy with policy makers to resolve the underlying problems (police raid in 2006 and funding interruption in 2014), were followed by resumption of outreach and services.

Between April and September 2016, outreach contacts and clinic visits declined again, when government funding was once more interrupted, and the number of peer educators, outreach workers and managers was reduced. Program indicators again returned to earlier levels when funding resumed. Recent changes in sex work—including new venues (private houses) and modes of solicitation (cell phones)—present new challenges but program performance, as measured by outreach contacts and clinic attendance, remains strong.

**Figure 4:**
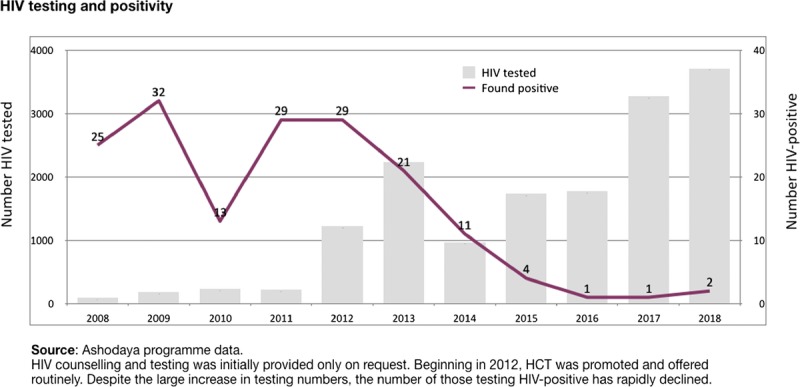
HIV testing and new HIV-positives 2008–2018.

The scale-up of HIV testing and counseling and ART services took place over several years in the middle period when overall program performance was strongest.^15,16^ After years of voluntary testing with low uptake, routine testing was promoted beginning in 2012, following government guidelines, and targets of 6 monthly testing were reached by 2013. Yet, these numbers fell by more than half in 2014 with the disruptions of peer outreach work, then increased again when funding was restored. The steadily declining number of new HIV-positives detected, despite nearly universal testing, is evidence of progress in eliminating new HIV infections in local sex work networks (Fig. 4).

For over 90% of sex workers in Mysore and Mandya, the emphasis has been on staying HIV-negative, with high retention in both community- and clinic-based prevention services. Despite high reported condom use, a feasibility assessment of preexposure prophylaxis (PrEP) conducted in 2013 to 2014 showed a high level of interest. The PrEP was then introduced in consultation with the community to ensure clear understanding, appropriate selection, regular support, and monitoring; data from the initial pilot phase documented very high retention and follow-up.^24^

**Figure 5:**
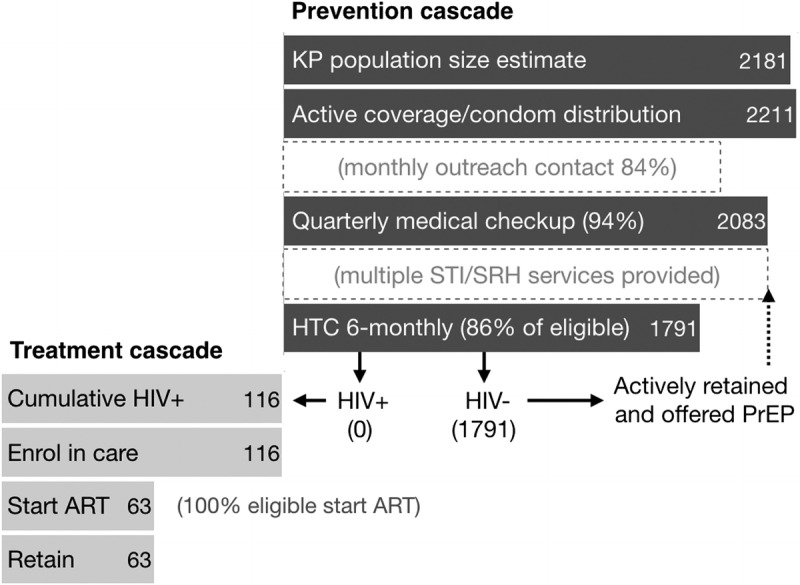
Ashodaya cascade performance, April through September 2017.

Access to quality care, support, and treatment also improved from 2012 driven by community need and demand, and facilitated by community structures.^16^ Antiretroviral treatment linkage and retention have exceeded 90% since 2013. Figure 5 presents routine program data covering the full prevention to care and treatment cascade for a recent 6-month period.

On the treatment side, cascade performance has remained strong with all identified positives linked to pre-ART or ART (data shown here reflect ART eligibility at CD4 > 350. India adopted test and treat in September 2017). Although 86% of those eligible underwent testing, no HIV-positives were detected during this period. Cumulative HIV-positives remained at 116 and all were enrolled in care. Peer support has enabled near complete retention in pre-ART or ART services over recent years, with few patients lost to death or migration. Viral load testing was not available during this period.

In the wider Mysore community, sentinel surveillance data show an 8-fold decline in HIV prevalence among ANC attendees, from 2% in 2005 to 0.25 in 2015.^25^

## DISCUSSION

Years of attention to universal access, with general population targets, have tended to divert attention away from sex workers, men who have sex with men, transgender persons, and people who use drugs. Focus is returning to these key populations, and their importance in STI/HIV transmission, regardless of epidemic stage, is receiving renewed attention.^26^ Unfortunately, this attention, and related funding for programs, is often narrowly tied to performance in HIV testing and linking HIV-positives to treatment.

Yet, cascade targets, and the largely general population approaches linked to them, do not automatically translate into effective key population strategies. In some scenarios driven mainly by HIV treatment cascade metrics, strong key population programs are actually penalized when their effective prevention efforts result in low “yield” of new HIV-positives who can then be started on ART.^27^ Worse, narrow service-oriented approaches and targets may have unintended effects on programs and key population communities, weakening prevention and undermining community participation and resilience.

In contrast, communities of sex workers across India have learned to interrupt STI/HIV transmission directly while achieving excellent program performance across cascades. Building on early work of Durbar Mahila Samanwaya Committee in the Sonagachi area of Kolkata, sex workers in Mysore replicated, innovated, and adapted to their own context, to interrupt STI/HIV transmission, ensure high treatment uptake and retention, and tackle a range of health and social problems affecting their community. Lessons from these experiences can inform effective key population programming elsewhere.^9,10,13–16,28^

First, both program and survey data support early and rapid control of HIV/STI transmission following effective interventions in “upstream” sex work networks. High uptake and utilization of basic condom and STI interventions, promoted actively and frequently through peer networks, had measurable impact on sex worker HIV and STI rates locally. This in turn would be expected to reduce “downstream” transmission in the general population, and there is supportive evidence for this in steep HIV prevalence declines among ANC attendees from 2005 to 2015.^24^ Models from other Indian sites with strong community-based interventions have estimated similar outcomes.^29^

Second, the vigorous community response that rallied around early STI/HIV prevention efforts facilitated introduction, uptake, and utilization of new interventions and services, with high retention rates and measurable public health impact. Microplanning strengthened outreach, whereas STI screening and PPT helped control curable STIs.^15,18,30^ Such experiences built a sense of “collective agency,” confidence, and capacity to address other problems, from violence to human trafficking.

Third, Ashodaya's strong platform of community-based clinical services also facilitated introduction of HIV-specific services. Antiretroviral treatment improved life expectancy and quality for HIV-positive sex workers, whereas PrEP was successfully introduced to provide additional protection for those HIV-negative. Microplanning, regular medical checkups, and regular program data review have enabled the community to monitor and support high uptake, utilization, and retention for optimal outcomes. Analyzed together, data on cascade performance—linked to population-level outreach and service utilization by both HIV-negative and HIV-positive sex workers—is far more complete than limited data from stand-alone HIV-testing programs elsewhere.

Fourth, Ashodaya, like other CBO implementing HIV programs, is vulnerable to disruptions in funding. When that occurs, most programs are unable to quantify the effects of service disruptions. Ashodaya's strong programmatic monitoring, on the other hand, enabled it to relate interruptions in basic community interventions (outreach contacts, condom distribution) and clinical services (regular checkups), to evidence of increasing transmission (STIs). After almost 10 years of strong programming, the absence of symptomatic STIs at regular checkups suggested very low sexual transmission risk. STIs returned quickly when services were disrupted and remained somewhat higher even after the regular checkups resumed.

Yet, outreach and services were restored, and there is growing evidence that sexual transmission has indeed slowed to near-elimination levels within local sex work networks. This is supported by strong cascade data linked to population denominators through microplanning. In such a context, the shrinking numbers of new HIV-positives detected with nearly universal HIV testing and counseling, linkage and retention in care and treatment, provide solid evidence for elimination of both new infections and morbidity/mortality related to HIV and other STIs.

The main limitation of this retrospective observational study is its reliance on programmatic data, supplemented only intermittently by more rigorous population-based surveys. However, the magnitude of trends suggests that biases were minimal, and the changes were real. This is especially true since 2009–2012, after the introduction of microplanning, when both outreach contacts and clinic visits reached near saturation levels with respect to the estimated sex worker population. By 2013, quarterly screening was almost universal, clinically detectable STIs had virtually disappeared and HIV had started declining toward local elimination.

Ashodaya's experience and processes contain lessons for key population communities elsewhere. The community centrality of program design, adoption of community-led processes, capacity building of community members to monitor and analyze data locally, and to use it for local decision making, have all contributed to success. Ashodaya served as a learning site under Avahan to catalyze rapid scale-up of critical community-led processes to other sites. “Ashodaya Academy,” a sex worker-led training and research center supported by UNAIDS, serves as a global learning site to disseminate and adapt core principles and innovations.^22,31^

## Supplementary Material

SUPPLEMENTARY MATERIAL
