# Supplementary figures and images for: Sex Worker Community-led Interventions Interrupt Sexually Transmitted Infection/Human Immunodeficiency Virus Transmission and Improve Human Immunodeficiency Virus Cascade Outcomes: A Program Review from South India
Source: Sex Transm Dis. 2019 Jul 11;46(8):556–62. doi: 10.1097/OLQ.0000000000001020 (PMC6629169; doi:10.1097/OLQ.0000000000001020)

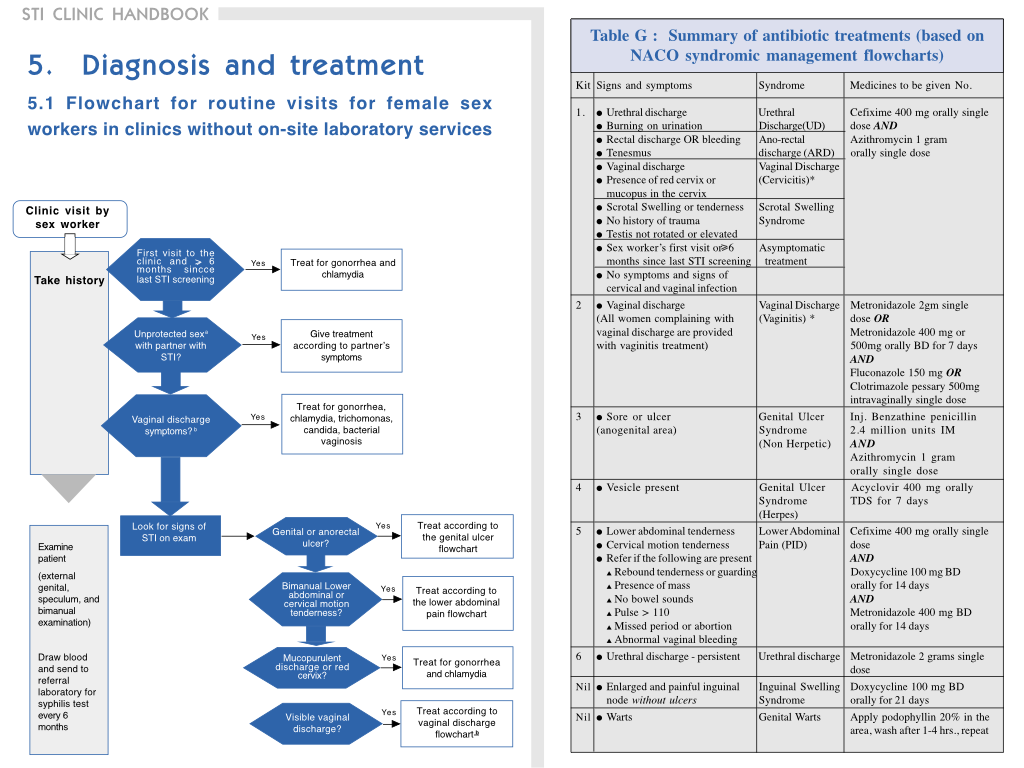

Supplement: SUPPLEMENTARY MATERIAL [file olq-46-556-s001.tiff]
